# Residential exposure to motor vehicle emissions and the risk of wheezing among 7-8 year-old schoolchildren: a city-wide cross-sectional study in Nicosia, Cyprus

**DOI:** 10.1186/1476-069X-9-28

**Published:** 2010-06-18

**Authors:** Nicos Middleton, Panayiotis Yiallouros, Nicolaos Nicolaou, Savvas Kleanthous, Spiros Pipis, Maria Zeniou, Philip Demokritou, Petros Koutrakis

**Affiliations:** 1 Department of Nursing, School of Health Sciences, Cyprus University of Technology, Nicosia, Cyprus; 2 Cyprus International Institute for the Environment and Public Health in association with Harvard School Public Health, Cyprus University of Technology, Limassol, Cyprus; 3 Respiratory Research Group, School of Translational Medicine, Wythenshawe Hospital, Manchester, UK; 4 Air Quality Section, Department of Labor Inspection, Nicosia, Cyprus; 5 Department of Environmental Health, Exposure, Epidemiology & Risk Program, Harvard School of Public Health, Boston MA 02215, USA

## Abstract

**Background:**

Several studies have reported associations between respiratory outcomes in children and a range of self-reported, administrative or geographical indicators of traffic pollution. First-time investigation into the frequency of asthmatic symptoms among 7-8 year-old Cypriot children in 1999-2000 showed increased prevalence in the capital Nicosia compared to other areas. Geographical differences on an island the size of Cyprus may reflect environmental and/or lifestyle factors. This study investigates the relationship between self-reported symptoms and residential exposure to motor vehicle emissions among Nicosia schoolchildren.

**Methods:**

The addresses of children in the metropolitan area of Nicosia who participated in the original survey (N = 1,735) were geo-coded and the level of exposure of each child was assessed using distance- and emission-based indicators (i.e. estimated levels of particulate matter and nitrogen oxides emissions due to motor vehicles on main roads around the residence). Odds ratios of wheezing and asthma diagnosis in relation to levels of exposure were estimated in logistic regression models adjusting for person-based factors, co-morbidity and intra-school clustering.

**Results:**

We found an increased risk of wheezing at distances less than 50 m from a main road and/or only among those experiencing the highest levels of exposure. The strongest effect estimates were observed when exposure was defined in terms of the cumulative burden at all roads around the residence. Adjusted odds ratios for current wheezing were 2.33 (95% CI 1.27, 4.30) amongst the quartile of participants exposed to the highest levels of PM at all roads 50 m of their residence and 2.14 (95% CI 1.05, 4.35) for NOx, with no effect at intermediate levels of exposure. While the direction of effect was apparent at longer distances, differences were generally not statistically significant.

**Conclusions:**

Children experiencing the highest burden of emissions in Nicosia seem to be at a higher risk of reporting asthmatic symptoms. Due to the small number of children residing at close proximity to main roads and lack of evidence of risk at intermediate levels of exposure or longer distances, the observed pattern alone does not explain the generally higher prevalence observed in urban Nicosia compared to other areas.

## Background

In the last three decades there have been significant increases in the prevalence as well as severity of asthma and other allergic disorders among children worldwide [1]. The International Study of Asthma and Allergies in Childhood (ISAAC) questionnaire has been extensively used to describe the epidemiology of asthma internationally [2,3]. Estimates of asthma prevalence range from 30% in the UK and the USA to as low as 5% in Greece, indicating large differences across the industrialized world [3]. The prevalence of asthmatic symptoms in two out of five districts in Cyprus (Nicosia and Limassol) was investigated for the first time using the ISAAC questionnaire during the academic year 1999-2000 [4]. Amongst 7-8 year olds, the survey showed increased prevalence of wheezing in the metropolitan area of Nicosia compared to the rest of the study area (i.e rural areas in Nicosia and both urban and rural areas in Limassol) i.e. 9.0% Vs 5.8%. A 2003 pilot study of 128 15-year-old children also found increased allergic sensitization among those living in the city of Nicosia [5]. The relatively high level of homogeneity in the ethnic and genetic composition on an island the size of Cyprus would suggest that such geographical differences in the prevalence of asthmatic symptoms may reflect environmental and/or lifestyle factors.

Exposure to traffic pollution may contribute, if not to the development, at least to the exacerbation of asthma as well as a range of other adverse respiratory symptoms. While recently a number of cohort studies indeed suggest a link between early-life exposure and the development of asthma [6,7], most evidence on the association between exposure to traffic pollution and the experience of symptoms comes from cross-sectional or case-control studies. Using a variety of exposure measures, ranging from self-reported traffic density, proximity of residence to a main road or modeled estimates of motor vehicle emissions, such studies have commonly focused on children or susceptible populations such as the elderly. While it is not the intention of this study to systematically review the large body of evidence, it is reasonable to say that the majority of studies tend to report positive effects [8-10], while a smaller number of studies have reported no or little [11-14] or even negative effects [15]. Negative associations may simply reflect a selective migration of symptomatic subjects away from polluted areas, while in contrast, it has been argued that the use of self-reported indicators of exposure may tend to overestimate any association compared to more objective measures [16].

The observed higher prevalence of asthmatic symptoms in the capital of Cyprus may be partly due to an increased exposure to traffic pollution. Using information from the original 1999-2000 survey, this study mapped the location of residential addresses (as well as attending schools) of participating children living in the metropolitan Nicosia region (population approximately 270,000) and investigated the relationship between self-reported health status and exposure to motor vehicle emissions at the place of residence assessed using a range of (a) distance-based (i.e. proximity of residence to the nearest main road) as well as (b) emission-based indicators (i.e. estimates of particulate matter and nitrogen oxides due to motor vehicle emissions on roads within a pre-defined distance from the residence).

While most evidence on the association between the experience of asthma symptoms in children and exposure to traffic emissions comes from large cities in Europe and North America., similar evidence in the context of smaller cities or other parts of the world has been limited. The aim of this study was to investigate the relationship between asthma symptoms and residential exposure to traffic emissions in Nicosia, a relatively small city in the Eastern Mediterranean region where both geo-climatic conditions and outdoor activity patterns may differ considerably from other much larger European capitals. In addition, the study aimed at elucidating the extent to which any observed pattern of association could explain the overall higher prevalence of asthma recorded in Nicosia compared to the rest of the island.

## Methods

### Data and data sources

During the original survey between 30 September 1999 and 9 March 2000, the Greek-version of the core ISAAC questionnaire was distributed to all second-grade primary schoolchildren (ages 7-8) at the school setting and was completed at home by consenting parents [4]. The support of the educational authorities ensured a participation rate of 81.2%. In addition to self-reported asthmatic and other allergic symptoms (i.e. eczema and rhinitis) as well as potential risk factors (such as family history of allergy, parental smoking and pet ownership), residential addresses were also collected at the time. While it is true that parents who might worry about the effect of exposure to traffic pollution might exaggerate their children's health experiences, it should be noted that at the time of the original survey participants were not aware of the hypothesis investigated here. For the purposes of the current study, addresses in the urban areas of Nicosia were geo-coded to enable the calculation of distances of each child's place of residence to the nearest main road. In total, eight municipalities were considered, including three sub-urban areas in the south outskirts of the city due to their geographical contiguity with the main urban sprawl of Nicosia. Areas north of the UN buffer zone (occupied by Turkish troops) could not be included since the original survey did not cover areas not controlled by the Republic of Cyprus.

Geographical data are not yet routinely available in Cyprus as in the US or many European countries and use of Geographical Information Systems (GIS) in health research is still at its infancy. In fact, we are not aware of any other epidemiological study in Cyprus that has employed GIS. With lack of appropriate maps from public sources, digital maps of the street network were purchased from a private GIS company (TERRA Ltd, http://www.terra.gr), which in 2006 produced the first (and currently only) digitized vector maps of all Cypriot cities using a series of raster maps (dated 2002 to 2005 with scale ranging between 1:2,500 to 1:7,500, originally provided by the Cyprus Cartography Branch of the Department of Land and Surveys) as well as employing 20 ground-teams to geo-reference and inform these maps accordingly with the use of GPS (Global Positioning Systems). While the resulting vector maps (scale 1:5,000, precision < 3 m) incorporated a characterization of roads into major arteries or residential streets, no other traffic-related information was available (e.g. volume or type of traffic, number of car lanes or density of buildings alongside the road).

Levels of particulate matter (PM) and nitrogen oxides (NOx) due to motor vehicle emissions along major roads in the city were obtained from the Air Quality Section (Cyprus Ministry of Labor), which in 2001 (around the same point in time as the original health survey) performed in collaboration with the University of Stuttgart the first systematic emissions inventory on the island from different linear, point and area sources, such as the hotel industry, agricultural activities and petrol stations [17]. For the road transport sector, the inventory used the CORINAIR framework proposed by the European Environment Agency to estimate emissions in member countries based on the car fleet composition, traffic load, average speed and length of each road sector and is expressed in grams of pollutant per period of time per length of road (more info: http://www.eea.europa.eu/publications) [18]. Hindered by the lack of information on the technology of the registered car fleet on the island, certain modifications or assumptions were necessary for the application of the COPERT methodology in Cyprus. These included the re-categorization of all passenger cars manufactured between 1986 and 1991 as non-catalytic (since unleaded gasoline was only introduced in Cyprus in 1992) and the assumption of geographical uniformity in the composition of the car fleet. Due to lack of other routine sources of traffic related data in Cypriot cities as well as the retrospective nature of this study to allow collection of self-reported information from the participants, these data allowed the calculation of exposure assessment measures in addition to proximity.

It should be noted that while the street network maps were produced at a more recent point in time than the data collection (and may thus represent changes that occurred since the survey e.g. new roads which appeared since or were re-classified into main roads), only the subset of roads included in the 2001 Emissions Inventory were considered in the analyses more closely representing the point in time when the health questionnaire was administered. Finally, to assist in the effective presentation of maps, additional background maps with administrative information such as municipality boundaries or topographical information such as the UN buffer zone were obtained from the German-based GIS company GfK GeoMarketing (http://www.gfk-geomarketing.com).

### Outcome and exposure assessment

Based on responses to the ISAAC questionnaire, three outcomes were investigated: (i) current wheezing (i.e. in the past 12 months), (ii) history of wheezing (i.e. ever having wheezed) and (iii) having ever had an asthma diagnosis. Reported symptoms of hay fever or eczema as well as a family history of atopy (i.e. among siblings or parents) were considered as co-morbidity. In addition to calculating the actual distance of each participant's residence to the nearest main road, a series of traffic exposure indicators were calculated by assigning to each child the estimated levels of PM or NOx emissions at (a) the road nearest to the residence, (b) the road within 150 m of the residence where emission levels were highest, and (c) the cumulative levels of emissions at all roads at a predefined distance of the residence. Rather than restricting the calculation to the nearest or the road with the highest levels, the cumulative measure is the sum of estimates at all road that cross the specified buffer and it does not represent actual total mass within that buffer. Due to their crude nature, these measures were only used as categorical rather than continuous variables in order to classify participants into increasing levels of exposure. As dispersion models indicate that levels of pollutants decrease exponentially to reach a plateau at distances longer than 150 m of a main road [19], circular buffer-zones of 50, 100 and 150 m radius were considered. A buffer zone of 300 m radius was further applied to represent a "safe zone" and, thus, describe a measure of baseline risk. The prevailing wind direction or speed were not considered. Consistent with previous practice, distance weights were applied based on a Gaussian probability distribution that assumes up to 95% decay of pollutants by 150 m from the source [12,20,21]. In other words, while a residence located on a main road was assigned the emissions as recorded on that road, a residence at 50 m of the same road would be assigned 70% of that value, dropping to 25% by 100 m and 5% at 150 m from the source. Finally, all exposure calculations were repeated for the school address and estimated levels were assigned to all children attending the same school.

### Statistical Analyses

The unadjusted prevalence of the three outcomes investigated here was calculated in relation to increasing proximity of the residence to the nearest main road (i.e. < 50 m, 50-100 m, 100-150 m, 150-300 m and > 300 m) or by levels of exposure (e.g. across quartiles of increasing levels of PM and NOx emissions). Evidence of differences in prevalence was assessed in Pearson χ^2^-tests while evidence for trend in proportions across ordered categories was assessed using an extension of the Wilcoxon rank-sum test (*nptrend *command in STATA). Multivariable logistic regression models were used to assess evidence of increased prevalence of symptoms by exposure indicators after adjusting for person-based risk factors (i.e. gender, nationality, birth weight, parental smoking, maternal smoking during pregnancy and pet ownership) as well as co-morbidity (i.e. personal history of hay fever or eczema as well as history of atopy among siblings or parents). In addition to parental history of atopy, we adjusted for personal history of hay fever and eczema in order to assess the possible confounding effect of allergic sensitization (also found to be more pronounced in the city of Nicosia than elsewhere) in the association between traffic pollution and asthma symptoms. Studies have shown associations between traffic exposure and allergic sensitization; even though the evidence is less consistent [7,22,23]. The possibility of over-adjusting (if, for instance, the direction of association between allergy and asthma is assumed to be causal) was assessed by the degree of attenuation in the effect estimates. Finally, since children living in the same area (or attending the same school) may be more likely to share environmental exposures and/or socio-economic experiences, models also adjusted for intra-cluster (either area or school) correlation to account for the possible lack of independence across individuals.

**Figure 1:**
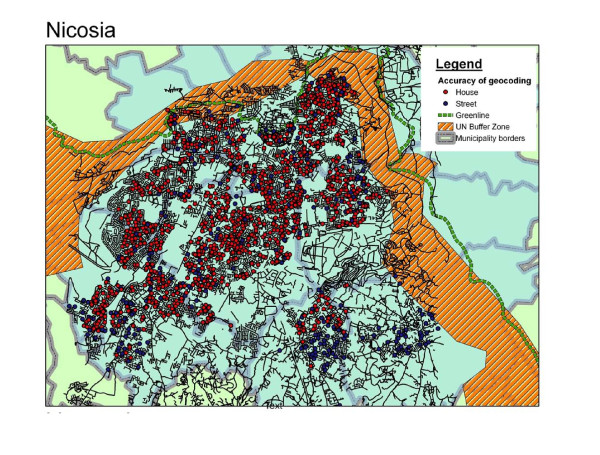
**Point map of participants' addresses in the city of Nicosia, Cyprus**. Geo-coded residential addresses of participants by level of accuracy of geo-coding showing (a) in red, those geo-coded accurately at house level and (b) in blue, those geo-coded at street level only.

Finally, sensitivity analyses assessed the effect of (a) excluding peripheral areas where the level of geographical information as well as the coverage of the emissions inventory was poorer (see Figure 1), (b) excluding participants whose addresses were not accurately geo-coded at the house level and (c) after also considering exposure at the school setting explicitly. In the absence of accurate time-activity information, exposure was assumed to be a weighted average of the exposure at home and at school with the same weights applied to all children reflecting the expected time spent in each setting (i.e. 6 hours a day at the school). Geo-coding and all geographical calculations were performed in ArcView 9.2. Statistical analyses were performed in STATA SE 9.0 (Stata Corporation, College Station, Texas).

## Results

Amongst 2,617 children who participated in the original survey from the district of Nicosia, 1,917 children (73%) resided in metropolitan areas and were included in this study. These children attended 48 different schools with an average number of 40 recruited students (inter-quartile range: 29-48). Ranging from no one reporting symptoms in three schools to a maximum of 27% in an inner-city school (IQR: 5-12% across schools), the prevalence of current wheezing averaged to 8.8% in the city of Nicosia. As many as 21.9% of the participants reported having wheezed in the past and 12.9% having been diagnosed with asthma while 29.3% of the participants reported family history of atopy. Along with estimates of all respiratory outcomes, Table 1 summarizes the socio-demographic characteristics of the participants. The majority (91.9%) were born in Cyprus, 48.9% were boys and in 46.4% of them at least one parent was a smoker.

**Table 1:** Participants characteristics, outcomes and levels of exposure (a) among all participants, (b) among those whose addresses were and were not geo-coded successfully and (c) among those whose addresses were geocoded at house versus at street level only

		**A. All**	**B. By geocoding success**			**C. By level of accuracy of geocoding**		
		(N = 1,917)	**Successful** (N = 1,735)	**Unsuccessful** (N = 182)	**P-value^1^**	**House** (N = 1,336)	**Street** (N = 399)	**P-value^1^**
	
**Characteristics**								
	
Male		48.9%	49.1%	47.8%	0.75	49.5%	47.6%	0.52
	
Born in Cyprus		91.9%	92.4%	87.3%	0.02	92.6%	91.7%	0.54
	
At least one parent not Cypriot		16.2%	12.5%	17.7%	0.05	12.1%	13.8%	0.37
	
Birth weight (mean in kg)		3.24	3.24	3.24	0.98	3.24	3.25	0.62
	
At least one parent smoker		46.4%	46.7%	45.1%	0.67	47.0%	45.7%	0.66
	
Smoking at least a pack/day		33.5%	33.5%	34.1%	0.69	34.1%	31.7%	0.63
	
Smoking during pregnancy		2.5%	2.5%	2.8%	0.80	2.4%	2.5%	0.90
	
Cat at home		3.4%	3.6%	2.2%	0.33	3.8%	3.1%	0.48
	
**Symptoms**								
	
Currently wheezing		8.8%	9.3%	7.3%	0.37	9.6%	8.3%	0.43
	
Ever having wheezed		21.9%	22.9%	20.1%	0.40	23.2%	21.7%	0.53
	
Ever had asthma diagnosis		12.9%	13.4%	13.0%	0.87	13.5%	13.2%	0.87
	
Personal history of eczema		8.2%	8.9%	5.1%	0.09	8.7%	9.4%	0.70
	
Personal history of hay fever		3.9%	4.5%	1.7%	0.10	4.6%	3.7%	0.50
	
Family history of atopy		29.3%	31.6%	26.9%	0.20	32.1%	30.2%	0.51
	
**Exposure (mean)**								
	
Distance to nearest main road (metres)			176.4			177.1	174.4	0.84
	
PM at nearest (kg per km per day)			0.22			0.26	0.21	0.15
	
NOx at nearest (kg per km per day)			4.60			4.52	5.10	0.23
	
Cumulative PM (kg per km per day)	within 50 m		0.40			0.39	0.42	0.16
		
	within 100 m		0.54			0.54	0.54	0.83
		
	within 150 m		0.78			0.80	0.71	0.12
		
Cumulative NOx (kg per km per day)	within 50 m		6.60			6.52	6.88	0.23
		
	within 100 m		9.46			9.51	9.29	0.73
		
	within 150 m		14.00			14.42	12.67	0.12
		

^1^p-values of χ^2^-test for independence or t-test for differences in means as appropriate.

The addresses of 1,735 children (90% of all) were geo-coded successfully. Table 1 also presents a comparison of the frequency of outcomes as well as socio-economic characteristics between those participants whose addresses were geo-coded successfully and the 10% of the participants who were further excluded. Although differences were not statistically significant, respiratory symptoms appeared slightly elevated in those whose addresses were geo-coded successfully, perhaps suggesting a higher interest in the survey among parents whose children experience symptoms. With the exception of children not born in Cyprus or to Cypriot parents (perhaps, also less likely to provide accurate address information), there didn't seem to be any differences in the characteristics between participants whose addresses were and were not geo-coded successfully.

Furthermore, two degrees of geo-coded accuracy were identified and treated accordingly: (a) cases where the full address was available i.e. both street and house number (N = 1,336, 77% of addresses) and (b) cases where due to inaccurate or incomplete information only the street was identified, in which case the residence was randomly assigned on that street (N = 399, 23%). Figure 1 shows the study area and plots the location of all geo-coded addresses showing in red the addresses that were geo-coded accurately at the house level and in blue those assigned at street level only. Accuracy appeared much poorer in the sub-urban municipalities in the south outskirts of the city. Although, there didn't seem to be any differences in participant characteristics, reported symptoms or levels of exposure in terms of accuracy of geo-coding (also see *Table *1), the effect of excluding participants whose addresses were not accurately geo-coded at house level was further assessed in sensitivity analyses.

**Figure 2:**
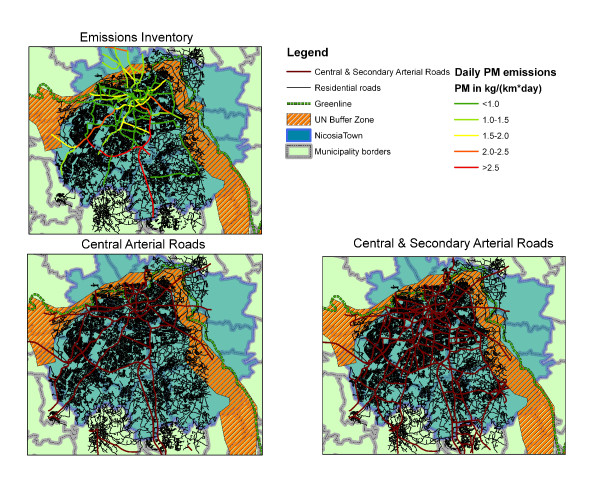
**The street network in the city of Nicosia, Cyprus**. The network of central arterial, secondary arterial and residential roads in the city of Nicosia along with the subset of roads covered in the Emissions Inventory depicting levels of PM emissions (ranging from lowest shown in green to highest shown in red).

Figure 2 shows the extent of the street network in the city of Nicosia as well as the subset of roads for which estimated levels of emissions were available from the Emissions Inventory. The figure shows levels of particulate matter due to motor vehicle emissions depicting roads with the lowest levels in green while roads with the highest levels in red. Levels of NOx were available for the same set of roads. The study area was restricted to the Greek-controlled areas (south of the UN buffer zone) since the original survey did not cover Turkish-Cypriot schools. Calculation of emissions-based indicators was restricted to the sub-set of roads that were included in the inventory. In general, the inventory covered the vast majority of roads officially characterized as central as well as some secondary traffic arteries but appeared less comprehensive in peripheral sections of the city. The effect of excluding areas with limited coverage of the emissions inventory was further assessed in sensitivity analyses.

As expected, the prevalence of symptoms in this age group (as well as asthma diagnosis) was statistically significantly higher in boys. Significant associations were observed between all three outcomes and maternal smoking during pregnancy, which generally persisted after adjusting for the effect of all other participant characteristics in multivariable models; for instance, in the case of asthma diagnosis the adjusted odds ratio was 2.2 (95% CI: 1.1, 4.7). Only small or no associations were observed with other potential risk factors, such as parental smoking and cat ownership while negative associations were observed with regards to the nationality of the child's parents but reasons why this should be a protective factor are unclear. Personal history of hay fever and family history of atopy were by far the strongest predictors of current wheeze with adjusted odds ratios of 4.1 (95% CI: 2.3, 7.4) and 2.9 (95% CI: 2.0, 4.2) respectively. This was also true in the case of history of wheezing and asthma diagnosis.

Those who reported current wheezing (N = 156) lived on average at 149.6 metres (95% CI: 119.9, 186.6) of a main road versus 178.7 m (95% CI: 167.9, 190.2) in the case of those who did not report symptoms (p-value of t-test for difference in means = 0.09). At 11.7% (95%CI: 7.6, 15.8), an increased prevalence of current wheezing was observed only among those residing within 50 m of a main road (N = 238), with no evidence of a stepwise association with proximity. At longer distances the prevalence was confined to levels similar to the overall prevalence observed in the city of Nicosia. The figures were 8.7% (95%CI: 5.2, 13.3) among participants whose residence was 50-100 m of a main road, 8.6% (95%CI: 5.1,13.5) among those within 100-150 m of a main road, 9.0% (95%CI: 6.4,12.1) among those within 150-300 m against a background prevalence of 9.0% (95%CI: 6.9,11.5) among participants whose residence was more than 300 m of a main road. Nevertheless, further stratifying participants into those who live within 50 m of main roads with high (N = 90, roads in orange and red colour on Figure 1) and low levels (N = 148) of PM, prevalence of current wheezing was estimated at 14.4% (95% CI: 7.9%, 23.4%) and 10.1% (95% CI: 5.7%, 16.1%) respectively versus 8.9% (95% CI: 7.5, 10.5) at longer distances (p-value for trend = 0.09). Similarly, for history of ever wheezing, these figures were 30.0%, 25.0% and 22.2% respectively (p-value for trend = 0.07) while for asthma diagnosis the figures were 22.5%, 10.3% and 13.2% respectively (p-value for trend = 0.11).

Table 2 presents the prevalence and odds ratios (and 95% CI) of current wheezing (a) by distance and (b) across increasing levels of distance-weighted PM and NOx emissions at the street nearest to the residence or at the street within 150 m of the residence where levels were highest. Adjusted odds ratios are presented (i) as estimated in models adjusting for gender and maternal smoking during pregnancy - the factors other than co-morbidity with the strongest associations, (ii) after further adjusting for co-morbidity and all other participant characteristics (except birth-weight for which information was unavailable for a large number of participants). While consistently higher odds of wheezing were observed among those in the highest exposure category, any statistical evidence of increased risk was generally restricted at distances of 50 m of a main road or the quartile of participants at the highest levels of PM exposure. While not statistically significant, at least the magnitude of the estimates did not attenuate after adjusting for confounding factors in multivariable models. Similar associations were observed for the other two outcomes. No clear associations were observed with levels of NOx.

**Table 2:** Prevalence and odds ratios (and 95% CI) of current wheezing by distance-based and emissions-based indicators of exposure before and after adjusting for person-based risk factors and co-morbidity as well as intra-school correlation in multivariable logistic models

		**N**	**Prevalence %**	**Unadjusted (N = 1675)**	**Adjusted^1 ^(N = 1650)**	**Adjusted^2 ^(N = 1461)**
		
**A. Distance-based indicators**						
1. By proximity	> 300 m	622	9.0	1.00	1.00	1.00
		
	150-300 m	413	9.0	0.99 (0.62,1.59)	0.93 (0.57,1.54)	0.89 (0.52,1.50)
		
	100-150 m	195	8.6	0.97 (0.53,1.76)	0.89 (0.47,1.68)	1.01 (0.51,2.00)
		
	50-100 m	207	8.7	0.96 (0.55,1.67)	0.94 (0.54,1.63)	1.08 (0.61,89)
		
	< 50 m	238	11.7	1.35 (0.95,1.91)	1.27 (0.89,1.80)	1.30 (0.86,1.97)
		
					
		
2. Within 50 m of a main road	No	1437	8.9	1.00	1.00	1.00
		
	Yes	238	11.7	1.36 (1.05,1.78)	1.32 (1.00,1.74)	1.33 (0.93,1.89)
		
					
		
3. Within 50 m of a road, stratified by levels of PM	No	1437	8.9	1.00	1.00	1.00
		
	Yes, Low PM	148	10.1	1.15 (0.79,1.68)	1.10 (0.76,1.58)	1.10 (0.76,1.58)
		
	Yes, High PM	90	14.4	1.73 (1.06,2.81)	1.72 (1.04,2.84)	1.72 (0.92,3.20)
		
					
		
4. Within 50 m of a road, stratified by levels of NOx	No	1437	8.9	1.00	1.00	1.00
		
	Yes, Low NOx	126	11.8	1.38 (0.94,2.03)	1.31 (0.90,1.91)	1.31 (0.88,1.93)
		
	Yes, High NOx	112	11.6	1.34 (0.82,2.20)	1.33 (0.81,2.21)	1.35 (0.75,2.43)
		
					
		
**B. Distance-weighted emissions-indicators^3^**						
		
1. PM at nearest street (kg/km per day)	None	622	9.0	1.00	1.00	1.00
		
	Lowest (< 0.5)	748	8.8	0.98 (0.68,1.41)	0.92 (0.63,1.35)	0.97 (0.67,1.41)
		
	Medium (0.5-1)	215	9.7	1.09 (0.72,1.66)	1.02 (0.68,1.54)	1.05 (0.67,1.65)
		
	Highest (> 1)	90	14.4	1.71 (1.00,2.90)	1.65 (0.96,2.81)	1.67 (0.82,3.38)
		
	P-value for trend			0.12	0.22	0.26
		
					
		
2. PM at street with highest levels (kg/km per day)	None	622	9.0	1.00	1.00	1.00
		
	Lowest (< 0.5)	757	8.7	0.97 (0.67,1.39)	0.91 (0.62,1.33)	0.95 (0.65,1.37)
		
	Medium (0.5-1)	202	10.3	1.17 (0.77,1.78)	1.09 (0.72,1.65)	1.13 (0.72,1.75)
		
	Highest (> 1)	94	13.8	1.62 (0.96,2.75)	1.57 (0.92,2.67)	1.65 (0.84,3.25)
		
	P-value for trend			0.11	0.20	0.20
		
					
		
3. NOx at nearest street (kg/km per day)	None	622	9.0	1.00	1.00	1.00
		
	Lowest (< 10)	725	8.5	0.95 (0.64,1.39)	0.89 (0.59,1.33)	0.92 (0.62,1.37)
		
	Medium (10-20)	230	11.6	1.34 (0.88,2.06)	1.27 (0.83,1.95)	1.35 (0.85,2.14)
		
	Highest (> 20)	98	11.2	1.28 (0.74,2.20)	1.23 (0.71,2.14)	1.19 (0.58,2.47)
		
	P-value for trend			0.19	0.31	0.31
		
					
		
4. NOx at street with highest levels (kg/km per day)	None	622	9.0	1.00	1.00	1.00
		
	Lowest (< 10)	813	9.7	1.09 (0.78,1.53)	1.02 (0.72,1.46)	1.08 (0.76,1.53)
		
	Medium (10-20)	135	6.6	0.72 (0.36,1.47)	0.70 (0.35,1.41)	0.71 (0.32,1.59)
		
	Highest (> 20)	105	11.4	1.30 (0.76,2.25)	1.23 (0.70,2.18)	1.21 (0.61,2.42)
		
	P-value for trend			0.75	0.94	0.91

^1^Adjusted for gender and smoking during pregnancy, ^2^Also adjusting for co-morbidity (i.e. eczema, hay fever and family history of atopy) and all other risk factors except birthweight, ^3 ^Cut-off points for levels of exposure were defined based on the 50% and 75% (i.e. highest quartile) of the distribution of non-zero values.

Stronger effect estimates were observed in terms of cumulative burden of emissions around the residence. Nevertheless, there was again strong evidence of non-linearity and any association appeared to be mainly driven by a twofold increase in risk concentrated only at the quartile of participants who experience the highest levels of exposure. Table 3 presents unadjusted and fully adjusted odds ratios for all three outcomes investigated across increasing levels of cumulative PM and NOx emissions burden at all roads within 50, 100 and 150 m from the residence. In each case, cut-off points for the highest category of exposure were defined based on the 75^th ^percentile (i.e. highest quartile) of the distribution of non-zero values (i.e. among those within the specified distance from a main road), while when a middle category appears, this represents the 50^th ^percentile. Adjusted odds ratios for current wheezing amongst the quartile of participants who experience the highest levels of PM within a 50 m radius from their residence was 2.33 (95% CI: 1.27, 4.30) as compared to those who reside further away (N = 1440) and 2.14 (95% CI: 1.05, 4.35) for levels of NOx. Other than slightly larger standard errors, the magnitude of effect remained largely unaffected in multivariable models. Excluding hay fever and eczema from the models does not alter inferences, suggesting that the observed association between traffic pollution and current wheeze is independent of the presence of hay fever or eczema. While we tested for (and found no) evidence of effect modification by allergic status, the study was under-powered to address this issue properly. Associations of a similar magnitude were observed for the other two outcomes - history of wheezing and asthma diagnosis. Perhaps with the exception of history of wheezing which displayed more of a stepwise relationship with exposure, there was no evidence of increased risk among those who experience intermediate levels of exposure. While the direction of effect was also apparent at longer distances, associations were commonly short of statistical significance.

**Table 3:** Unadjusted and adjusted odds ratios (and 95% CI) of reported symptoms (current or ever wheeze) and asthma diagnosis across increasing levels of cumulative exposure to PM or NOx emissions at all roads 50, 100 and 150 m from the residence

		**Unadjusted (N = 1675)**	**Adjusted^1 ^(N = 1461)**	**Unadjusted (N = 1675)**	**Adjusted^1 ^(N = 1460)**	**Unadjusted (N = 1671)**	**Adjusted^1 ^(N = 1463)**
		
		**Current wheezing**		**Ever having wheezed**		**Ever asthma**	
Cumulative PM at 50 m	> 50 m, None	1.00	1.00	1.00	1.00	1.00	1.00
		
	< 50 m, Low	1.17 (0.80,1.73)	1.06 (0.66,1.70)	1.29 (0.89,1.88)	1.30 (0.83,2.04)	0.96 (0.56,1.66)	0.84 (0.45,1.57)
		
	< 50 m, High^2^	1.97 (1.23,3.16)	2.33 (1.27,1.30)	1.32 (0.83,2.09)	1.66 (0.91,3.01)	1.78 (0.97,3.25)	2.51 (1.36,4.64)
		
							
		
Cumulative PM at 100 m	> 100 m, None	1.00	1.00	1.00	1.00	1.00	1.00
		
	< 100, Low	1.00 (0.68,1.46)	1.08 (0.72,1.63)	1.16 (0.86,1.56)	1.24 (0.89,1.72)	0.97 (0.66,1.40)	0.85 (0.56,1.29)
		
	< 100, High^2^	1.86 (1.17,2.97)	1.92 (1.06,3.46)	1.29 (0.85,1.95)	1.41 (0.94,2.38)	1.72 (1.02,2.90)	2.00 (1.11,3.51)
		
							
		
Cumulative PM at 150 m	> 150 m, None	1.00	1.00	1.00	1.00	1.00	1.00
		
	< 150, Low	0.98 (0.64,1.50)	1.05 (0.66,1.67)	1.00 (0.72,1.38)	1.10 (0.74,1.62)	0.88 (0.58,1.33)	0.83 (0.52,1.32)
		
	< 150, Medium	0.98 (0.61,1.57)	1.19 (0.71,1.97)	1.02 (0.67,1.57)	1.17 (0.74,1.82)	0.88 (0.52,1.47)	0.97 (0.57,1.62)
		
	< 150, High^2^	1.58 (0.91,2.73)	1.60 (0.79,3.22)	1.58 (1.13,2.21)	1.51 (0.95,2.41)	1.29 (0.81,2.05)	1.31 (0.77,2.22)
		
	P-value for trend	0.20	0.16	0.05	0.10	0.67	0.61
		
							
		
Cumulative NOx at 50 m	> 50 m, None	1.00	1.00	1.00	1.00	1.00	1.00
		
	< 50 m, Low	1.12 (0.79,1.57)	1.08 (0.64,1.54)	1.17 (0.84,1.61)	1.24 (0.84,1.85)	1.02 (0.63,1.64)	0.96 (0.58,1.59)
		
	< 50 m, High^2^	2.12 (1.26,3.58)	2.14 (1.05,4.35)	1.71 (1.07,2.73)	1.83 (0.94,3.57)	1.58 (0.86,2.87)	1.90 (0.94,3.82)
		
							
		
Cumulative NOx at 100 m	> 100 m, None	1.00	1.00	1.00	1.00	1.00	1.00
		
	< 100, Low	1.01 (0.71,1.44)	1.11 (0.75,1.63)	1.10 (0.83,1.47)	1.19 (0.86,1.63)	0.96 (0.67,1.37)	0.85 (0.57,1.27)
		
	< 100, High^2^	1.78 (1.06,2.96)	1.76 (0.93,3.30)	1.49 (0.98,2.26)	1.61 (0.95,2.71)	1.72 (0.96,3.09)	1.89 (1.00,3.57)
		
							
		
Cumulative NOx at 150 m	> 150 m, None	1.00	1.00	1.00	1.00	1.00	1.00
		
	< 150, Low	1.01 (0.69,1.48)	1.14 (0.75,1.71)	1.01 (0.75,1.28)	1.14 (0.81,1.61)	0.89 (0.60,1.31)	0.89 (0.58,1.37)
		
	< 150, Medium	0.90 (0.47,1.72)	1.12 (0.57,2.19)	0.99 (0.71,1.54)	1.09 (0.58,2.03)	0.76 (0.37,1.58)	0.84 (0.39,1.78)
		
	< 150, High^2^	1.56 (0.97,2.52)	1.44 (0.75,2.74)	1.58 (0.96,1.69)	1.47 (1.01,2.15)	1.38 (0.83,2.27)	1.27 (0.72,2.26)
		
	P-value for trend	0.20	0.25	0.04	0.12	0.59	0.71

^1^Adjusted for gender, smoking during pregnancy, co-morbidity and all other risk factors except birthweight, ^2 ^Cut-off points for levels of exposure were defined based on the 50% (in the case of the medium category) and/or 75% (i.e. highest quartile for the high category) of the distribution of non-zero values.

**Figure 3:**
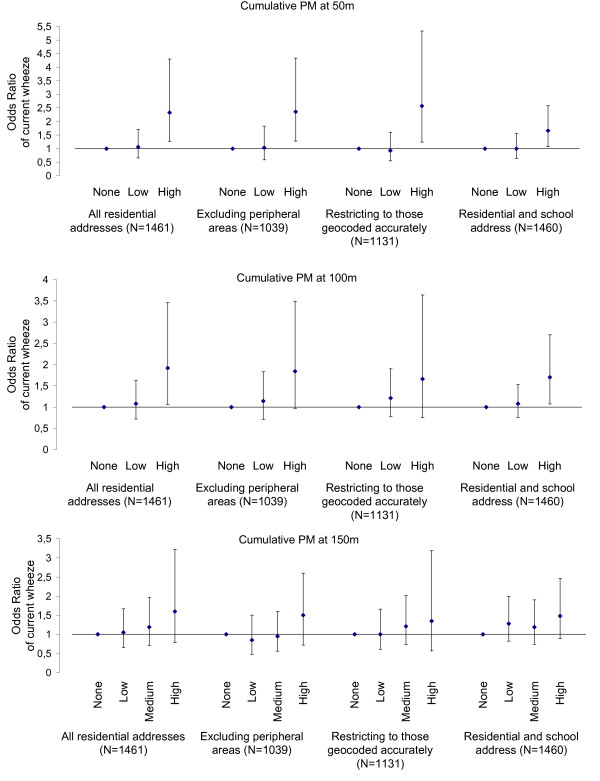
**Sensitivity analyses for the effect estimates across increasing levels of PM emissions**. Adjusted odds ratios (and 95% CI) of current wheezing across no, low and high (i.e. quartile of participants at the highest exposure) levels of cumulative exposure to PM emissions on roads at 50, 100 and 150 m from the residence before and after (a) excluding peripheral areas, (b) restricting to those addresses geocoded accurately and (c) considering exposure at the school location.

**Figure 4:**
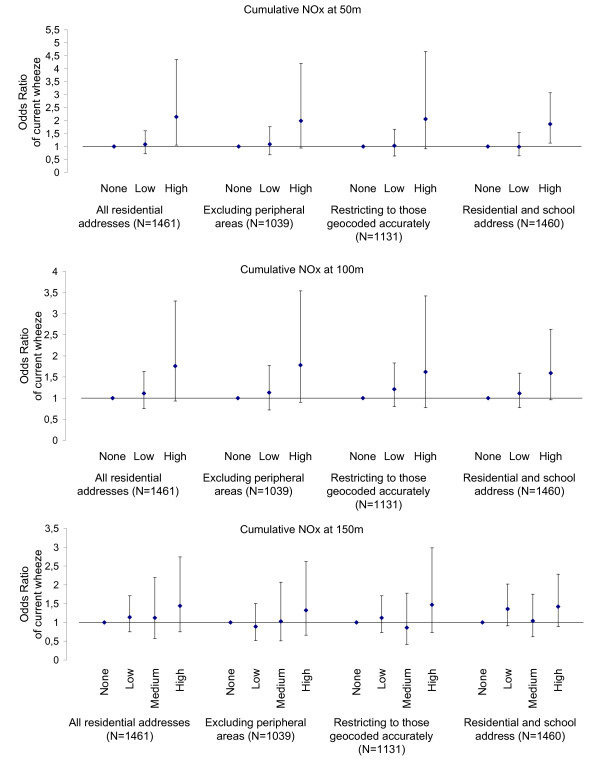
**Sensitivity analyses for the effect estimates across increasing levels of NOx emissions**. Adjusted odds ratios (and 95% CI) of current wheezing across no, low and high (i.e. quartile of participants at the highest exposure) levels of cumulative exposure to NOx emissions on roads at 50, 100 and 150 m from the residence before and after (a) excluding peripheral areas, (b) restricting to those addresses geocoded accurately and (c) considering exposure at the school location.

Both the direction and magnitude of effects were largely unaffected after excluding peripheral areas (where the emission inventory was less comprehensive) or restricting the analysis to those children whose addresses were geocoded accurately at house level. Results of the sensitivity analyses with regards to the observed associations between current wheezing and PM or NOx emissions are presented in Figures 3 and 4 respectively. When exposure at the school setting was also considered, effect estimates were generally weaker. Nevertheless, tighter confidence intervals were observed (and as a result statistical significance was preserved) due to the much larger number of children considered exposed at this enlarged definition of exposure. For instance, adjusted odds ratios for current wheezing amongst the quartile of participants who experience the highest cumulative levels of PM within a 50 m radius from their residence and/or school was 1.66 (95% CI: 1.07, 2.58) compared to those who reside further away and 1.86 (95% CI: 1.13, 3.07) for levels of NOx, with no evidence of increased risk at intermediate levels of exposure. Inferences were similar in the case of the other two outcomes.

Finally, gender differences were not statistically significant to allow any inference about effect modification, even though consistent with the literature [24], the effect appeared much stronger in girls. Adjusted odds ratios for wheezing among girls (N = 740) were 1.00, 1.21 (95%CI 0.48, 3.09) and 2.96 (95%CI 1.18, 7.39) across increasing levels of cumulative PM exposure at 50 m while these figures were 1.00, 0.97 (95%CI 0.54, 1.74) and 1.85 (95%CI 0.71, 4.85) in boys (N = 693); p-value of likelihood ratio test for effect modification = 0.44.

## Discussion

### Main Findings

Consistently increased prevalence of symptoms and asthma diagnosis was observed in those who reside close to main roads and/or experience the highest levels of exposure. Effect estimates were statistically significant when the cumulative burden was considered. Up to 2-fold increases in risk for all symptoms were observed among those experiencing the highest cumulative levels of PM or NOx emissions at roads 50 metres of their residence after adjusting for person-based risk factors and co-morbidity. Nevertheless, there was no evidence of an increased risk at intermediate levels of exposure. While the direction of effect was apparent at longer distances, differences were generally not statistically significant.

### Strengths - Limitations

Since the study is cross-sectional in nature, it can not pinpoint to traffic pollution as a risk factor for the development of wheezing or asthma but only describe the association between the experience of symptoms at different levels of exposure. The participation of children in the original survey was very high and reflected the true prevalence and spatial distribution of the asthmatic population of this age group in the city of Nicosia. Exposure was defined in terms of distance or levels of emissions at the surrounding roads. Despite the limitations of the available data and the crude nature of exposure assessment, the consistency in the direction and pattern of effect estimates across the different definitions of exposure (i.e. proximity or emissions based) is reassuring. While symptoms were self-reported, parents were not obviously aware of the hypothesis investigated here at the time of the original survey. Furthermore, associations were not restricted to current status but were also apparent for asthma diagnosis, generally considered to be a more reliable measure.

The lesser degree of geo-coded accuracy of some participants' addresses introduces misclassification in exposure; however, any error should be random (i.e. it is not expected to systematically over- or under-estimate exposure) and non-differential (i.e. there is no reason why it should relate to the outcome) and would thus only be expected to underestimate the true effect. Furthermore, there was no evidence of differences in participant characteristics, symptoms or exposure indicators by level of geo-coding accuracy. Furthermore, inferences were largely unaffected when the analyses were repeated to exclude either addresses geo-coded less accurately or peripheral areas.

With current lack of area-based socio-economic indicators in Cyprus and with no person-based measures of household income or education available from the original health survey, models did not control for a possible confounding effect of socio-economic factors. Socio-economic gradients in levels of traffic pollution vary greatly across different study areas. For example, while a study in urban areas in Netherlands and Munich found no differences in exposure by parental education as a measure of social class [25], in sharp contrast, a recent study in Rome found that levels of air pollution were in fact higher in areas of higher socio-economic status [26]. The relatively recent expansion of Nicosia as well as continuing development of central areas in the city has commonly resulted in areas with a mix of households of varied socio-economic standing side by side. While it is true that buildings located on major roads may attract families of lower socioeconomic status, higher exposure to traffic pollution in that case may be viewed as a direct cause and as such, perhaps part of the causal chain in any relationship between low socio-economic status and the experience of symptoms.

### Traffic emissions and the risk of wheezing

Increases in the prevalence of asthma worldwide have occurred over a short period of time to be explained by only genetic changes, suggesting that environmental risk factors may be the underlying cause. A second Cypriot survey, preformed nearly 8 years after the first, has just been completed and analyzed indicating that asthma in this age group is on the rise on the island. A range of lifestyle and environmental factors have been implicated including lack of exposure to infections and microbial products in early life, changes in dietary habits, physical activity, sun exposure and traffic pollution [27,28]. Exposure to traffic pollutants have been shown to produce a range of respiratory symptoms, especially among susceptible populations [24,29], or even interact with environmental triggers such as pollen to precipitate symptoms in allergic subjects [30,31]. While evidence on the association between traffic exposure and allergic sensitization is less consistent, in a multi-centre case-control study in France, it was found that early-life exposure to traffic pollution is associated with asthmatic symptoms even after adjusting for both personal and parental allergy [9].

A number of studies have investigated residential exposure to traffic pollution using a similar geographical perspective in cities much larger than Nicosia. Using outcomes as diverse as lung function [14,32], hospital admissions [33], clinically diagnosed asthma [11,20], or commonly self-reported symptoms as in this case [26], studies have previously produced some mixed results. However, evidence of positive associations is accumulating with several studies now reporting dose-response relationships with exposure commonly up to 150 m from major roads [8,9,20,34,35], or at least among those experiencing the highest levels of exposure [22,36], while some still report no or little effect [14]. In the urban environment of Nicosia, symptoms appeared elevated only within 50 m of a main road and/or only among those at the highest levels of exposure across all definitions used. While, generally, no effect was observed at intermediate levels of exposures or longer distances, the extent to which this is a product of the small size of the study, misclassification in (or the crude nature of) the exposure, or even the use of single outcome self-reported measure is not clear. While the observed pattern persists even after excluding peripheral areas or inaccurately geo-coded addresses, the nature of the study does not permit inferences about the shape of the exposure response function. Nevertheless, it is important to draw attention to the synergistic role of proximity and exposure; for example, no effect was observed at distances further than 50 m or even among those residing within 50 m of main roads with low estimated levels of PM emissions. Yet, when exposure was defined in terms of cumulative emissions, an effect was still apparent at somewhat longer distances (i.e. 100 m) at least among those experiencing the highest levels of exposure. It is also important to highlight that, unlike current symptoms or asthma diagnosis, history of wheezing displayed a somewhat more stepwise relationship with exposure. While this could not be formally tested here, this may be suggestive of symptoms, albeit less frequent, at lower levels of exposure.

### Exposure assessment

Previous studies have employed a wide range of exposure indicators, including self-reported traffic density [37], distance to motorways, major roads or heavily travelled roads [11,38,39], census data on car or truck traffic [40,41], or model estimates of emissions [22,42]. It has often been argued that subjective assessment tends to overestimate true exposure [16,25]. Recently, however, a multicentre research study in Italy performed a validation study whereby self-reported traffic density was compared to actual traffic flow measurements as well as checked for internal consistency among participants in the same census block. While authors appreciate that their findings may not necessarily apply to other locations or population, the study showed that the observed association between asthma symptoms in 13-14 year old children and parent-reported traffic density was not caused by reporting bias among parents whose children experience symptoms [43]. Lastly, defining exposure simply as proximity takes no account of type and density of traffic, vehicle speed and periods of acceleration all of which affect emission levels [44]. Studies have started to combine concentration measurements from different sites to represent the spatial variability of air pollution at exact residential locations [6,7]. With only two monitoring stations in the city of Nicosia this was not possible.

Evidence on the validity of any of the previously used indicators, including emission- or concentrations-based measures, as an estimate of actual personal exposure is still limited [45,46]. Children do not spent time only at home but also at school, play areas, parks etc as well as being exposed to several indoor sources of air pollutants. With no information on indoor exposures (other than parental smoking) or time-activity patterns, the measures used here certainly do not represent personal exposure. Some justification for the use of residential proximity as a proxy for personal exposure in epidemiological studies comes from a recent study which showed that children living near busy roads have higher personal exposures that children living further away even after adjusting for indoor sources of exposure [47].

Finally, any cross-sectional measure does not represent past exposures. Generally though, more is known about the experience of symptoms from similar studies with a cross-sectional design than the contribution of long-term exposure. Interestingly, a recent case-control study of 4-14 year-old children used an index of lifelong exposure at home and school addresses since birth and only found an association restricted to the first three years of life [9]. Recently, there has also been evidence from cohort studies that early-life exposure to traffic pollution may also directly contribute to the development of asthma [6,7,20,24]. With no information on residential history or the period of residence at the current address, it was not possible to investigate this issue.

## Conclusions

Using both proximity as well as distance-weighted estimated levels of motor vehicle emissions on main roads around the residence in a city-wide survey of 7-8-year old children in the capital of Cyprus, this study showed that at least those who reside at close proximity to a main road were more likely to have reported symptoms or having had an asthma diagnosis. The effect appeared to be mainly driven by a twofold increase in risk concentrated among those participants who experience the highest levels of emissions, with no much evidence of risk at intermediate levels of exposure or longer distances. The observed pattern and the small number of children residing at this close proximity to main roads would not explain the overall higher prevalence of symptoms observed in Nicosia, compared to other areas on the island.

## List of abbreviations

CI: Confidence Intervals; GIS: Geographical Information Systems; m: metres; IQR: Interquartile range; NOx: Nitrogen Oxides; OR: Odds Ratio; PM: Particulate Matter.

## Competing interests

The authors declare that they have no competing interests.

## Authors' contributions

PY, NN and SP formed the Pediatric Respiratory Research group that designed the original survey. PY and PK conceived and secured funding for the current study. SK had originally designed and performed the emissions inventory and which he kindly provided for the purposes of this study. PY, MZ and NM gathered and compiled the data. NM performed the geographical and statistical analyses and wrote the first draft of this paper. All authors assisted in the interpretation of results and contributed towards the final version. All authors read and approved the final manuscript.
